# A clinical prediction model for lung metastasis risk in osteosarcoma: A multicenter retrospective study

**DOI:** 10.3389/fonc.2023.1001219

**Published:** 2023-02-10

**Authors:** Shengping Zheng, Longhao Chen, Jiaming Wang, Haosheng Wang, Zhaohui Hu, Wanying Li, Chan Xu, Minmin Ma, Bing Wang, Yangjun Huang, Qiang Liu, Zhi-Ri Tang, Guanyu Liu, Tingting Wang, Wenle Li, Chengliang Yin

**Affiliations:** ^1^ Department of Orthopedics, Xianyang Central Hospital, Xianyang, China; ^2^ Faculty of Orthopaedics and Traumatology, Guangxi University of Traditional Chinese Medicine, Nanning, China; ^3^ Department of Orthopedics, The First Affiliated Hospital of Harbin Medical University, Harbin, China; ^4^ Department of Orthopaedics, The Second Hospital of Jilin University, Changchun, China; ^5^ Department of Spinal Surgery, Liuzhou People’s Hospital, Liuzhou, China; ^6^ Clinical Medical Research Center, Xianyang Central Hospital, Xianyang, China; ^7^ School of Physics and Technology, Wuhan University, Wuhan, China; ^8^ Faculty of Medicine, Macau University of Science and Technology, Macau, Macau SAR, China; ^9^ State Key Laboratory of Molecular Vaccinology and Molecular Diagnostics & Center for Molecular Imaging and Translational Medicine, School of Public Health, Xiamen University, Xiamen, China

**Keywords:** nomogram, SEER, osteosarcoma, lung metastasis, webpage calculator

## Abstract

**Background:**

Lung metastases (LM) have a poor prognosis of osteosarcoma. This study aimed to predict the risk of LM using the nomogram in patients with osteosarcoma.

**Methods:**

A total of 1100 patients who were diagnosed as osteosarcoma between 2010 and 2019 in the Surveillance, Epidemiology and End Results (SEER) database were selected as the training cohort. Univariate and multivariate logistic regression analyses were used to identify independent prognostic factors of osteosarcoma lung metastases. 108 osteosarcoma patients from a multicentre dataset was as valiation data. The predictive power of the nomogram model was assessed by receiver operating characteristic curves (ROC) and calibration plots, and decision curve analysis (DCA) was utilized to interpret the accurate validity in clinical practice.

**Results:**

A total of 1208 patients with osteosarcoma from both the SEER database(n=1100) and the multicentre database (n=108) were analyzed. Univariate and multivariate logistic regression analyses showed that Survival time, Sex, T-stage, N-stage, Surgery, Radiation, and Bone metastases were independent risk factors for lung metastasis. We combined these factors to construct a nomogram for estimating the risk of lung metastasis. Internal and external validation showed significant predictive differences (AUC 0.779, 0.792 respectively). Calibration plots showed good performance of the nomogram model.

**Conclusions:**

In this study, a nomogram model for predicting the risk of lung metastases in osteosarcoma patients was constructed and turned out to be accurate and reliable through internal and external validation. Moreover we built a webpage calculator (https://drliwenle.shinyapps.io/OSLM/) taken into account nomogram model to help clinicians make more accurate and personalized predictions.

## Introduction

Osteosarcoma is the most common malignant tumor of primary bone other than tumors of the lymphohematopoietic system. It is thought to originate from mesenchymal tissue, accounting for approximately 16% of primary bone tumors and 19% of malignant bone tumors, 90% of which are common osteosarcoma (1, 2). Some osteosarcoma patients were diagnosed with Lung metastasis (LM) at presentation, and about 50% of patients without LM at the beginning of the presentation were also developed LM during chemotherapy.

LM has a high mortality rate at an early stage and it can be mistaken for trauma or growing pains and neglected at the onset. In addition, severe side effects such as myelosuppression, gastrointestinal toxicity and hypersensitivity reactions and resistance to conventional chemotherapy greatly reduce the quality of life of patients (3, 4). Although the treatment model of osteosarcoma has been improved from “amputation-based” to “preoperative neoadjuvant chemotherapy, surgical resection of tumor, and postoperative adjuvant chemotherapy” in recent years, the 5-year survival rate of patients with LM <30% (5, 6). So there has been a demand for further study on osteosarcoma, especially the problem of LM to improve the quality of life of patients and overall survival rate (5, 6).

In recent years, nomogram have been extensively used worldwide to generate the likelihood of clinical events through complex computational formulas (7, 8). With the help of nomogram, clinicians can assess the risks of clinical events and then design individual treatment plans, determine the use of adjuvant therapies, optimize treatment protocols and consider appropriate patient counseling (9). Little research is found about LM combined with nomogram in osteosarcoma-related studies. Considering the important role of LM in the prognosis of osteosarcoma, this study aimed to use a nomogram to identify patients with osteosarcoma at high risk of LM.

Studies based on relevant Surveillance, Epidemiology and End Results (SEER) databases can target larger populations in different regions than single-centre studies. The SEER database (https://seer.cancer.gov/) currently collects approximately 34.6% of tumour incidence and survival data from US population-based cancer registries. It is considered the definitive source of tumour information in the US source (10). We extracted osteosarcoma patient data from SEER to build a prediction model, and further collected patient data from four medical centers in different regions of China for external validation of the model. In order to make the model truly clinically useful, we dynamized the model through a web calculator.

## Methods

### Data sources and inclusion criteria

The population data for this retrospective study included all patients diagnosed with osteosarcoma. We extracted 1100 patients diagnosed with osteosarcoma between 2010 and 2016 from the SEER database using the SEER*STAT (8.3.5) software,which divided into a training set and an internal validation set in the ratio of 7:3. The inclusion criteria were as follows:(1) ICD-O-3 for osteosarcoma:9180-9185;(2) complete clinical information with a clear survival or follow-up time;(3) age >18 years. Exclusion criteria were as follows: (1) clinical information was missing or unknown; (2)other primary tumour disease and cases with unknown metastatic status.

Demographic and clinical characteristics in the SEER database were as follows: Race, Age, Sex, Primary site, Histological differentiation, Laterality, Grade, T-stage, N-stage, Surgery, Radiation, Chemotherapy, Bone metastases,and lung metastases. We also recruited 108 patients from multi-centre data, as external validation.The Osteosarcoma’s tumor node metastasis (TNM) stage was evaluated based on the 7th edition of the American Joint Committee on Cancer (AJCC) staging manual. “SEER Combined Mets at DX-lung (2010 +)” was used to identify the presence of lung metastasis in a newly diagnosed.Two experts from each hospital were independently responsible for acquiring and processing the data to avoid subjective bias, and The data was reconfirmed by a third person.

Finally, we used a Microsoft Excel tool (Microsoft Excel, 2013, Redmond, USA) to record all data information accurately and sort it according to date.

### Nomogram construction, validation and clinical application

A total of 1208 patients were identified by recording patient demographics and baseline clinical survey data, with 1770 patients as the training cohort, 330 patients an internal validation set from the SEER database and multicentre data as the validation dataset (n=108). Thereafter, all data were subjected to preliminary descriptive statistics to describe the baseline characteristics of the two cohorts. Baseline patient characteristics were compared between the training cohort and the validation cohort by chi-square test.

Firstly, we assessed variables predicting the occurrence of lung metastases in Osteosarcoma patients by univariate logistic regression analysis. Subsequently, multivariate logistic regression analysis was used to assess each variable at the 0.05 significance level and to identify independent factors associated with lung metastasis. Thirdly, the data for the training cohort was used to construct column plots based on these prognostic factors Univariate and multivariate logistic regression models were used to identify variables significantly associated with pulmonary metastases from Osteosarcoma in the SEER dataset. Columnar line graphs of lung metastasis prediction based on independent factors were then constructed from the SEER cohort. In addition, we performed decision curve analysis (DCA) to assess the clinical utility and value of the line graphs. After building the nomogram, we evaluated a series of metrics. For calibration, we first assessed the discriminatory ability of the new model using the area under the time-related subject operating characteristic (ROC) curve (AUC) to improve the accuracy and comprehensiveness of the comparison (11), with the higher area under the curve (AUC) being more accurate. All analyses were performed using R software(version 4.0.5). Two-tailed p-values less than 0.01 were considered statistically significant. The relationship between actual and predicted probabilities was verified by calibration curves. The agreement between the predicted probabilities and the actual situation using column line plots was assessed by plotting calibration plots (12). Finally, we used DCA to assess the clinical validity of the model (13). This study statement confirms that all methods were performed in accordance with relevant guidelines and regulations, and all experimental protocols were approved by the Ethics Committee of Xianyang Central Hospital, confirming that informed consent was obtained from all subjects or their legal guardians.

### Statistical methods and software

In this study, normally distributed continuous variables such as Age and Survival time were expressed as mean ± standard deviation, while other categorical variables were expressed as numbers and percentages (N, %). SPSS statistical software (SPSS version 26.0, Chicago, USA) was used for the statistics and R software (version 4.0.5, http://www.Rproject.org) was used for the column line graphs development and evaluation. Firstly, univariate logistic regression analysis was used to identify risk factors associated with lung metastases, and patients with P<0.05 in univariate analysis were included in multivariate logistic regression analysis to identify independent prognostic factors. In addition, we calculated the corresponding 95% confidence intervals (95% CI). Based on the independent risk factors, column line plots predicting the development of lung metastases in patients with Osteosarcoma were constructed. Calibration plots were used to assess the accuracy of the columnar plots, while DCA curves were used to assess the clinical value of the columnar plots. Unlike sensitivity, specificity and area under the curve, DCA directly assesses the utility of clinical risk prediction models in decision making (14). p-values less than 0.05 were considered statistically significant.

## Results

### Baseline patient characteristics

A total of 1208 patients with Osteosarcoma were retrieved for further analysis based on inclusion criteria and screened for inclusion.1770 patients as the training cohort, 330 patients an internal validation set from the SEER database and 108 patients from the multicentre were included in the validation cohort. The training cohort was used to construct and internally validate the column line graphs, and the validation cohort was used for external validation. Detailed demographic information and clinical characteristics of the two cohorts and their comparisons are shown in **Table 1**. As shown in **Table 1**, there were no statistically significant differences between the SEER database and Multicenter cohorts in terms of Age, Survival time, Sex, Primary site, Differentiation, Grade, T stage, N stage, Surgery, Radiation, bone metastases and lung metastases (p>0.05), but there were significant differences in terms of race, laterality and chemotherapy (p< 0.05).

**Table 1:** Baseline data table of the training group and the validation group.

Variables	level	Overall(n=1208)	SEER data n=1100	Multicenter data n=108	p
Race (%)	black	164 (13.6)	164 (14.9)	0 (0.0)	<0.001
	other	218 (18.0)	110 (10.0)	108 (100.0)	
	white	826 (68.4)	826 (75.1)	0 (0.0)	
Age (mean (SD))	NA	33.00 (24.10)	33.18 (24.08)	31.13 (24.32)	0.399
times (mean (SD))	NA	30.18 (22.75)	30.12 (22.60)	30.81 (24.30)	0.764
Sex (%)	female	552 (45.7)	502 (45.6)	50 (46.3)	0.976
	male	656 (54.3)	598 (54.4)	58 (53.7)	
Primary.Site (%)	Axis bone	317 (26.2)	292 (26.5)	25 (23.1)	0.336
	Limb bone	790 (65.4)	713 (64.8)	77 (71.3)	
	other	101 (8.4)	95 (8.6)	6 (5.6)	
Grade (%)	Moderately differentiated	41 (3.4)	41 (3.7)	0 (0.0)	0.108
	Poorly differentiated	299 (24.8)	276 (25.1)	23 (21.3)	
	Undifferentiated; anaplastic	553 (45.8)	504 (45.8)	49 (45.4)	
	unknown	288 (23.8)	254 (23.1)	34 (31.5)	
	Well differentiated	27 (2.2)	25 (2.3)	2 (1.9)	
Laterality (%)	left	518 (42.9)	478 (43.5)	40 (37.0)	0.042
	Not a paired site	161 (13.3)	152 (13.8)	9 (8.3)	
	right	529 (43.8)	470 (42.7)	59 (54.6)	
Stage group (%)	I	198 (16.4)	181 (16.5)	17 (15.7)	0.811
	II	562 (46.5)	514 (46.7)	48 (44.4)	
	III	51 (4.2)	44 (4.0)	7 (6.5)	
	IV	285 (23.6)	259 (23.5)	26 (24.1)	
	UNK stage	112 (9.3)	102 (9.3)	10 (9.3)	
T (%)	T1	421 (34.9)	383 (34.8)	38 (35.2)	0.243
	T2	564 (46.7)	519 (47.2)	45 (41.7)	
	T3	41 (3.4)	34 (3.1)	7 (6.5)	
	TX	182 (15.1)	164 (14.9)	18 (16.7)	
N (%)	N0	1093 (90.5)	1001 (91.0)	92 (85.2)	0.144
	N1	37 (3.1)	32 (2.9)	5 (4.6)	
	NX	78 (6.5)	67 (6.1)	11 (10.2)	
surgery (%)	No	229 (19.0)	206 (18.7)	23 (21.3)	0.602
	Yes	979 (81.0)	894 (81.3)	85 (78.7)	
Radiation (%)	No	1060 (87.7)	959 (87.2)	101 (93.5)	0.078
	Yes	148 (12.3)	141 (12.8)	7 (6.5)	
Chemotherapy (%)	No	239 (19.8)	229 (20.8)	10 (9.3)	0.006
	Yes	969 (80.2)	871 (79.2)	98 (90.7)	
Bone metastases (%)	No	1144 (94.7)	1042 (94.7)	102 (94.4)	0.883
	unknown	7 (0.6)	6 (0.5)	1 (0.9)	
	Yes	57 (4.7)	52 (4.7)	5 (4.6)	
Lung metastases (%)	No	988 (81.8)	901 (81.9)	87 (80.6)	0.828
	Yes	220 (18.2)	199 (18.1)	21 (19.4)	

In **Table 2**, out of a total of 1208 patients with Osteosarcoma, 220 developed pulmonary metastases and 988 did not. There were significant differences between the LM and NO-LM groups in terms of Survival time, Stage group, T stage, N stage,Surgery and Bone metastases (p<0.001). In the LM group, 135 (61.4%) patients underwent surgery compared to 844 (85.4%) in the NO-LM group. A total of 148 (12.3%) patients received Radiation and 1060 (87.7%) patients did not receive Radiation. A total of 969 (80.2%) patients received chemotherapy and 239 (19.8%) patients did not receive chemotherapy. A total of 1144 (94.7%) patients did not develop bone metastases, compared to 7 (0.6%) and 57 (4.7%) patients with unknown and developing bone metastases respectively. We also compared LM and NO-LM groups in the training cohort, most similar results were also found in **Table S1**.

**Table 2:** Baseline data for patients presenting with and without lung metastases.

Variables	level	Overall(N=1208)	No(N=988)	Yes(N=220)	p
category (%)	Multicenter data	108 (8.9)	87 (8.8)	21 (9.5)	0.828
	SEER data	1100 (91.1)	901 (91.2)	199 (90.5)	
times (mean (SD))	NA	30.18 (22.75)	32.93 (22.97)	17.84 (16.99)	<0.001
Age (mean (SD))	NA	33.00 (24.10)	32.88 (23.70)	33.51 (25.83)	0.724
Sex (%)	female	552 (45.7)	471 (47.7)	81 (36.8)	0.004
	male	656 (54.3)	517 (52.3)	139 (63.2)	
Primary Site (%)	Axis bone	317 (26.2)	264 (26.7)	53 (24.1)	0.526
	Limb bone	790 (65.4)	639 (64.7)	151 (68.6)	
	other	101 (8.4)	85 (8.6)	16 (7.3)	
Grade (%)	Moderately differentiated	41 (3.4)	36 (3.6)	5 (2.3)	0.137
	Poorly differentiated	299 (24.8)	236 (23.9)	63 (28.6)	
	Undifferentiated; anaplastic	553 (45.8)	450 (45.5)	103 (46.8)	
	unknown	288 (23.8)	240 (24.3)	48 (21.8)	
	Well differentiated	27 (2.2)	26 (2.6)	1 (0.5)	
Laterality (%)	left	518 (42.9)	426 (43.1)	92 (41.8)	0.369
	Not a paired site	161 (13.3)	137 (13.9)	24 (10.9)	
	right	529 (43.8)	425 (43.0)	104 (47.3)	
Stage group (%)	I	198 (16.4)	194 (19.6)	4 (1.8)	<0.001
	II	562 (46.5)	550 (55.7)	12 (5.5)	
	III	51 (4.2)	50 (5.1)	1 (0.5)	
	IV	285 (23.6)	84 (8.5)	201 (91.4)	
	UNK stage	112 (9.3)	110 (11.1)	2 (0.9)	
T (%)	T1	421 (34.9)	382 (38.7)	39 (17.7)	<0.001
	T2	564 (46.7)	448 (45.3)	116 (52.7)	
	T3	41 (3.4)	25 (2.5)	16 (7.3)	
	TX	182 (15.1)	133 (13.5)	49 (22.3)	
N (%)	N0	1093 (90.5)	914 (92.5)	179 (81.4)	<0.001
	N1	37 (3.1)	24 (2.4)	13 (5.9)	
	NX	78 (6.5)	50 (5.1)	28 (12.7)	
surgery (%)	No	229 (19.0)	144 (14.6)	85 (38.6)	<0.001
	Yes	979 (81.0)	844 (85.4)	135 (61.4)	
Radiation (%)	No	1060 (87.7)	879 (89.0)	181 (82.3)	0.009
	Yes	148 (12.3)	109 (11.0)	39 (17.7)	
Chemotherapy (%)	No	239 (19.8)	205 (20.7)	34 (15.5)	0.091
	Yes	969 (80.2)	783 (79.3)	186 (84.5)	
Bone metastases (%)	No	1144 (94.7)	965 (97.7)	179 (81.4)	<0.001
	unknown	7 (0.6)	1 (0.1)	6 (2.7)	
	Yes	57 (4.7)	22 (2.2)	35 (15.9)	

### Univariate and multivariate logistic regression results

Risk variables for lung metastasis were analysed by applying univariate and multivariate logistic regression in patients with Osteosarcoma (**Table 3**). Univariate logistic models showed that significant factors such as Survival time, Sex, T stage, N stage, Surgery, Radiation, and Bone metastases were associated with lung metastasis. Further we used multivariate logistic regression analysis to confirm the association between T stage (T2, OR=2.337, 95% CI=1.517-3.601, p<0.001; T3, OR=4.197, 95% CI=1.727-10.199, p<0.01; Tx, OR=2.056, 95% CI=1.168-3.621, p< 0.05), N stage (NX, OR=2.110, 95% CI=1.133-3.928, p<0.05) and Bone metastases (yes, OR=4.329, 95% CI=2.265-8.275, p<0.001; unknown, OR=9.848, 95% CI=1.113-87.117, p<0.05) were independent risk factors, and Survival time (OR=0.973, 95% CI=0.963-0.983, p<0.001) and Surgery (yes, OR=0.540, 95% CI=0.355-0.822, p<0.01) were independent protective factors for lung metastases in patients with Osteosarcoma, and these factors were significantly associated with lung metastases in patients with Osteosarcoma.

**Table 3:** Univariate and multivariate logistic regression analysis of risk factors for lung metastasis in patients with osteosarcoma.

Variables	Univariate OR (95% CI)	p value	Multivariate OR (95% CI)	p value
Age (years)	1.003 (0.996-1.009)	0.402	/	/
Survival time (month)	0.961 (0.952-0.971)	<0.001	0.973 (0.963-0.983)	<0.001
Race				
White	1 (reference)	1 (reference)	1 (reference)	1 (reference)
Black	1.129 (0.738-1.728)	0.576	/	/
Other	1.099 (0.661-1.826)	0.716	/	/
Sex				
Male	1 (reference)	1 (reference)	1 (reference)	1 (reference)
Female	0.671 (0.490-0.920)	<0.05	0.636 (0.449-0.902)	<0.05
Primary site				
Limb bones	1 (reference)	1 (reference)	1 (reference)	1 (reference)
Axis of a bone	0.842 (0.587-1.209)	0.352	/	/
other	0.867 (0.491-1.532)	0.623	/	/
Grade				
Well differentiated	1 (reference)	1 (reference)	1 (reference)	1 (reference)
Moderately differentiated	0.210 (0.028-1.598)	0.132	/	/
Poorly differentiated	0.701 (0.260-1.891)	0.83	/	/
Undifferentiated; anaplastic	1.372 (0.885-2.128)	0.157	/	/
Unknown	1.127 (0.755-1.683)	0.558	/	/
Laterality				
Left	1 (reference)	1 (reference)	1 (reference)	1 (reference)
Right	1.095 (0.789-1.521)	0.588	/	/
Other	0.867 (0.528-1.422)	0.572	/	/
T				
T1	1 (reference)	1 (reference)	1 (reference)	1 (reference)
T2	2.445 (1.631-3.664)	<0.001	2.337 (1.517-3.601)	<0.001
T3	5.967 (2.757-12.915)	<0.001	4.197 (1.727-10.199)	<0.01
TX	3.645 (2.244-5.921)	<0.001	2.056 (1.168-3.621)	<0.05
N				
N0	1 (reference)	1 (reference)	1 (reference)	1 (reference)
N1	3.085 (1.479-6.434)	<0.01	1.555 (0.650-3.720)	0.321
NX	2.869 (1.694-4.860)	<0.001	2.110 (1.133-3.928)	<0.05
Surgery				
No	1 (reference)	1 (reference)	1 (reference)	1 (reference)
Yes	0.249 (0.177-0.350)	<0.001	0.540 (0.355-0.822)	<0.01
Radiation				
No	1 (reference)	1 (reference)	1 (reference)	1 (reference)
Yes	1.829 (1.251-2.752)	<0.05	1.250 (0.776-2.012)	0.359
Chemotherapy				
No	1 (reference)	1 (reference)	1 (reference)	1 (reference)
Yes	1.340 (0.897-2.003)	0.153	/	/
Bone metastases				
No	1 (reference)	1 (reference)	1 (reference)	1 (reference)
Yes	8.691 (4.850-15.574)	<0.001	4.329 (2.265-8.275)	<0.001
Unknown	24.160 (3.153-234.001)	<0.01	9.848 (1.113-87.117)	<0.05

### Nomogram construction and verification

**Figure 1:**
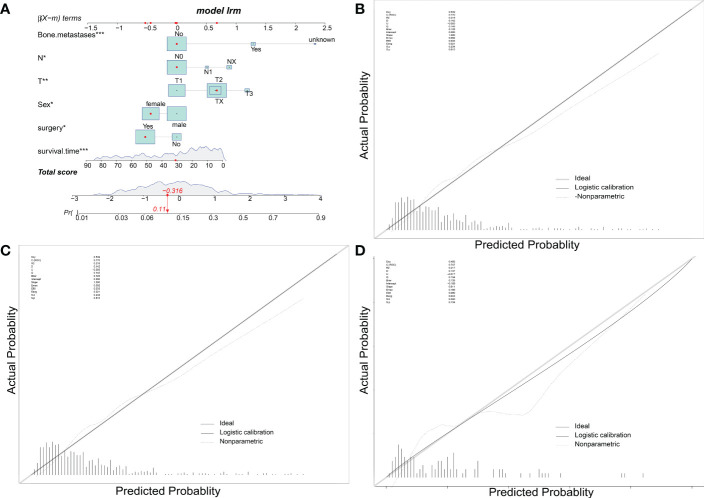
Nomogram predicts LM in patients with osteosarcoma. The LM Nomogram **(A)** contains six prognostic factors and illustrates the risk of LM in patients by mapping their values to covariate scales. The calibration curves for the prediction training group **(B)**, internal validation group **(C)** and multicentre validation group **(D)** are shown on the right.

**Figure 2:**
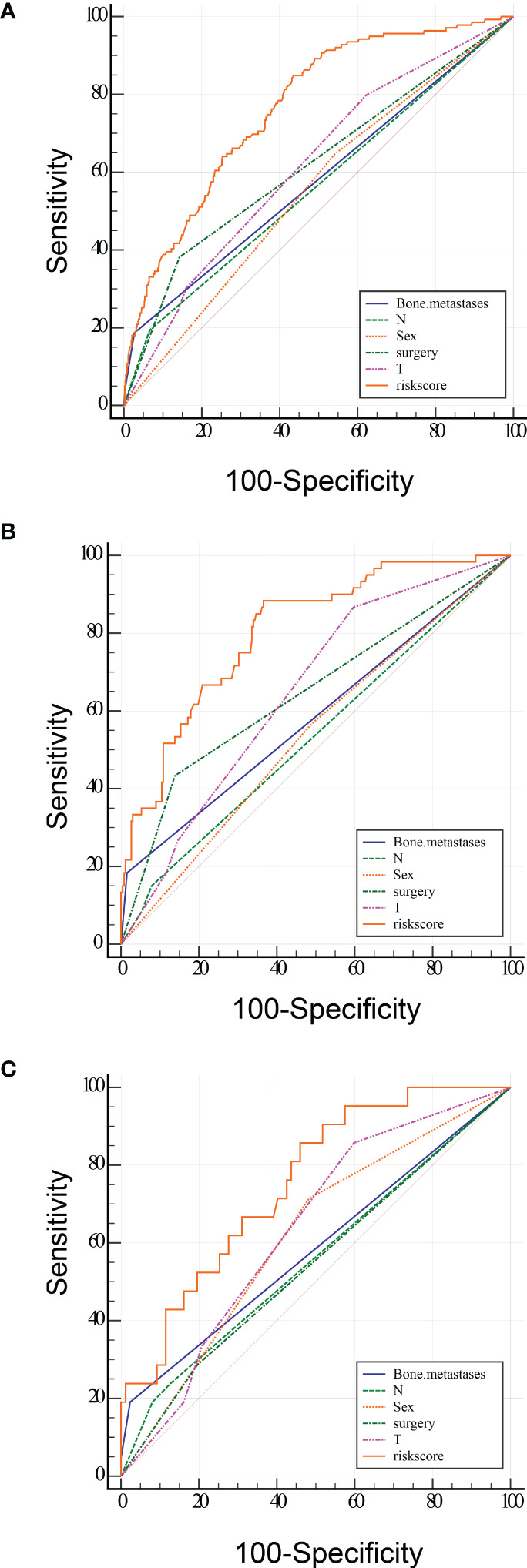
ROC curve analysis of the predicted LM nomogram (**A** training group, **B** internal validation group and **C** multicentre validation group) for indicating the discriminatory ability of the nomogram. The specific AUC values were shown in **Table 4**.

To predict the occurrence of lung metastasis in patients with Osteosarcoma, a nomogram was created based on the univariate and multivariate logistic regression results from the training set (**Figure 1A**). By counting each variable point, the total number of points may be correlated with the probability of lung metastasis in Osteosarcoma. As seen in **Figure 1A**, we found that bone metastases had the greatest effect on lung metastases, while surgery and sex having the least effect. The predicted nomogram was validated in both the SEER cohort and the multicentre cohort. The AUC is showed in Figure 2 A,and in internal validation and externalvalidation were 0.805 (95% CI: 0.758 to 0.846) and 0.753 (95% CI:0.661 to 0.831), respectively (**Figures 2B, C**). The calibration curve for the column line plot performed well in both the training and validation cohorts (**Figures 1B–D**). The results of the training and validation cohorts consistently showed that the predictive power of the nomogram was higher than that of the univariate factor.

In addition, we designed an online web-based calculator (https://drliwenle.shinyapps.io/OSLM/) to help us more effectively assess each patient’s risk of developing lung metastases, which can individually predict the probability of lung metastases based on the patient’s clinical characteristics. The results of the validation cohort showed that the new model significantly improves the accuracy and reliability of cancer prediction compared to the single factor in **Table 4**.

**Table 4:** AUC of training group and validation group.

Variables	SEER data (Training group)			SEER data (Internal validation group)			Multicenter data (External validation group)		
	AUC	SE	95% CI	AUC	SE	95% CI	AUC	SE	95% CI
Bone metastases	0.58	0.017	0.545 to 0.616	0.584	0.0255	0.529 to 0.638	0.584	0.0448	0.485 to 0.678
N	0.564	0.0176	0.528 to 0.599	0.535	0.0245	0.480 to 0.590	0.558	0.0517	0.460 to 0.654
Sex	0.553	0.0226	0.517 to 0.588	0.539	0.0357	0.483 to 0.594	0.616	0.0572	0.517 to 0.708
surgery	0.62	0.0218	0.584 to 0.654	0.648	0.0339	0.593 to 0.699	0.545	0.0548	0.447 to 0.641
T	0.617	0.0239	0.582 to 0.651	0.649	0.0329	0.595 to 0.701	0.634	0.0584	0.535 to 0.724
nomogram	0.767	0.0211	0.735 to 0.796	0.805	0.0303	0.758 to 0.846	0.753	0.0551	0.661 to 0.831

### Clinical application of LM nomogram

**Figure 3:**
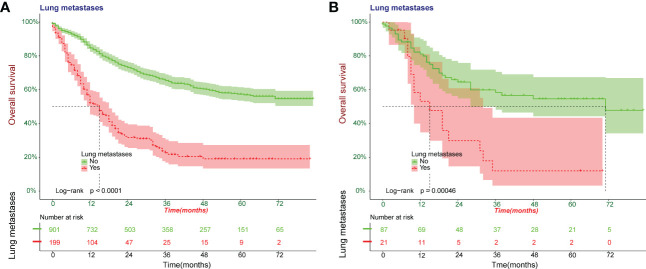
Kaplan-Meier survival curves were plotted for overall survival for patients with Osteosarcoma. A cohort of all patients was included to construct Kaplan-Meier survival curves **(A)**. Kaplan-Meier survival curves in external validation cohort **(B)**.

Kaplan-Meier survival curves were plotted for overall survival in 1208 patients (**Figures 3A, B**). The results showed that Osteosarcoma patients with lung metastases had significantly lower survival levels compared to the NO-LM group both in all patients and external validation cohort (p<0.0001).

**Figure 4:**
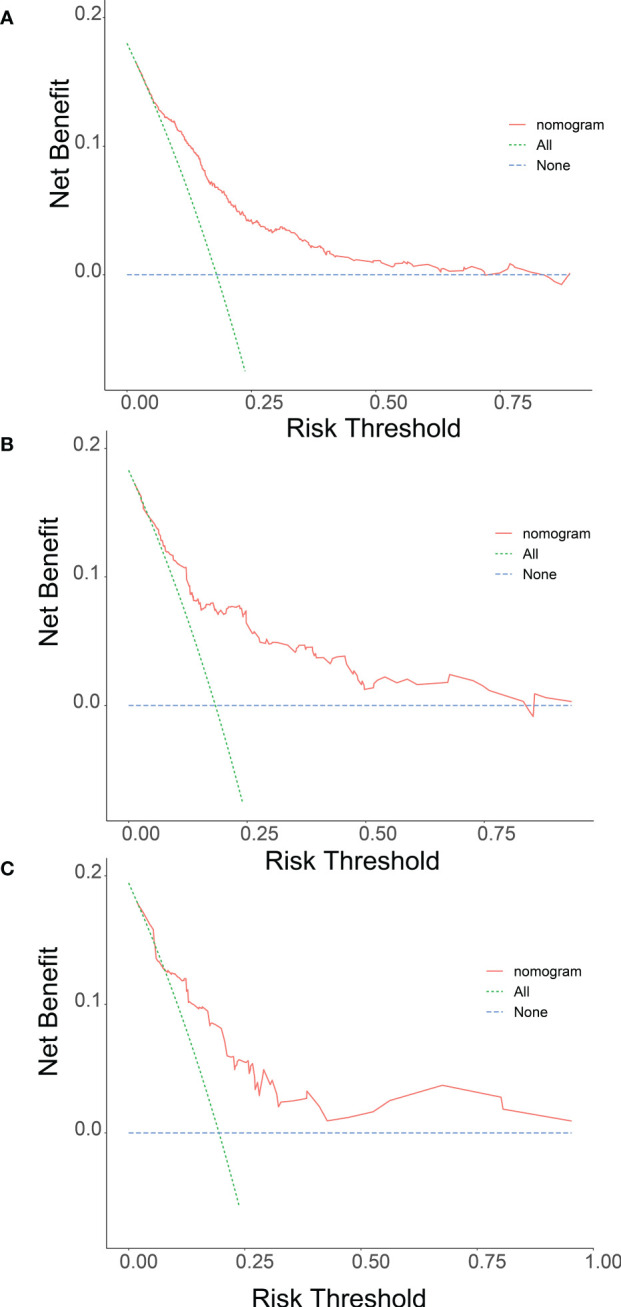
Nomogram determination curves (DCA) for lung metastasis risk. The red curve (number at high risk) indicates the number of people classified as positive (high risk) for each threshold probability for the Nomogram. The green curve (number at high risk with outcome) indicates the number of true positives at each threshold probability (**A** training group, **B** internal validation group and **C** multicentre validation group).

Meanwhile, we observed that the model has good clinical utility in predicting lung metastases in both the training cohort and the validation cohort of patients with Osteosarcoma. The net benefit of the training cohort was slightly higher than external validation cohort, which may be due to the limitation of the validation cohort size (**Figure 4**).

## Discussion

Currently, despite the availability of treatments such as surgery and chemotherapy, the 5-year survival rate of osteosarcoma patients was still less than 70% (15), and metastases were statistically observed in 30-40% of osteosarcoma patients (16), of which the lung was the most common site of involvement (17, 18), and the 5-year survival rate for developing LM was even lower than 30% (19). Therefore, screening patients with osteosarcoma at high risk of LM has significant implications for guiding medical decisions, both for clinicians and patients.

There have been some previous studies on clinical prediction models for osteosarcoma, but the data for both the training and validation groups of these articles were obtained from the SEER database and were not externally validated. Meanwhile, these studies were all predictive of survival or distal metastasis and did not predict lung metastasis, the most critical factor affecting the survival of osteosarcoma patients. In terms of model application, these studies also only did nomogram and did not make it visual and dynamic (20–24). Nomogram is a quantitative tool to assess risk and benefit, and has made an outstanding contribution to modern medical decision making (25). Compared with conventional prognostic factor analysis, such as regression analysis and correlation analysis, nomogram provided a personalized estimate of the probability of an event by combining various important factors, providing a clear picture for clinicians to predict the outcome of a disease so that they can make decisions that were more valuable for treatment in the clinical process (26). Nomogram approaches have better applications and more advantages in the interpretation of complex mathematical models compared with traditional statistical presentation. The ability of a single influencing factor to predict the prognosis of patients is limited. Therefore, we need to combine all clinically relevant factors as much as possible to play an active role in accurately predicting lung metastases in patients with osteosarcoma. In addition, a web-based calculator has been designed to make it more applicable for clinical work.

Our study showed T stage, N stage and bone metastases were the most important risk variables for lung metastases, which is also consistent with previous findings (27). The Osteosarcoma presenting patients with metastatic lesions have a low survival rate compared to patients with focal OS (28). All we can conclude is that both regional staging and bone metastases are associated with a higher risk of mortality after controlling for confounding factors. Unlike conventional tumours, Osteosarcoma may not be sufficiently sensitive to respond to chemotherapy and radiotherapy (29), such that surgery plays an important part in the treatment of OS. In our study, both survival time and surgery were independent protective factors and negatively correlated with lung metastases and it was demonstrated that patients without surgery had a poor prognosis. In contrast, the extent of surgical resection is important for local control of the tumour (30). However, in our study, the SEER database did not contain detailed data on the extent of surgical resection, so we did not specifically investigate the impact of this variable

From the Nomogram plot, bone metastasis was the best predictor for the occurrence of LM in this study. According to previous literature, the probability of bone metastasis in osteosarcoma was only 8-10%, but the occurrence of bone metastasis usually implies combined multi-metastatic disease (27, 30, 31). This phenomenon may be related to the skeletal microenvironment. The skeletal microenvironment includes osteocytes, bone marrow endothelium, adipocytes and the immune environment, structures that participate in bone homeostasis in an as-yet-undefined manner by strictly regulating skeletal physiology in order to meet the various needs of the host. However, once the skeletal microenvironment was disrupted, it became a favorable “ground” for tumor metastasis (32). It had been shown that the development of lung adenocarcinoma was associated with the activity of osteoblasts and the accumulation of tumor-promoting sialic acid binding Ig-like lectin (Siglec) F high neutrophils in the lung. The depletion of osteoblasts in this experiment was found to reduce neutrophil accumulation significantly and tumor growth, revealing an important link between resident cell populations in bone and tumor growth at primary and distant metastatic sites (33) The above study suggested that bone metastases were indeed more likely to be combined with metastases from other sites of tumor. Furthermore, the results of logistic regression analysis of the current study confirmed this idea, with the risk of LM in osteosarcoma patients with the presence of bone metastases (OR = 9.868) being 9.8 times higher than that of those without detected bone metastases. Therefore, it was reasonable to assume that osteosarcoma patients with bone metastases present had a greater risk of developing LM.

In addition, the occurrence of LM was also associated with regional lymph node metastasis, and according to the results of logistic regression analysis, the risk of LM was significantly higher in both those with regional lymph node involvement and those with unknown regional lymph nodes which was 3-4 times higher than that of osteosarcoma patients without lymph node metastasis, based on osteosarcoma patients without lymph node metastasis. This view has been supported by relevant studies, and an autopsy study performed by Hatori et al. confirmed that LM through the lymphatic route were more common in multifocal osteosarcoma (34). However, there was no clear explanation for how lymphatic metastasis occurs in osteosarcoma (35). It has been found that osteosarcoma metastases with the destruction of the ctorex, and it was suspected that the metastatic route may occur through the lymphatic vessels of the synovial membrane and bursa (36). In terms of osteosarcoma lymphatic metastasis detection, one study detected lymph node metastasis in pediatric sarcoma patients by whole-body imaging with positron emission tomography using 18-fluorodeoxyglucose (FDG) as a tracer (FDG-PET). Moreover, this method was also effective for the detection of bone metastases. Finally, it was worth noting that this study concluded that gender was also a risk factor for LM. The results of the logistic regression analysis showed that the risk of LM was higher in men than in women. As shown by the OR value (0.664), the male-to-female ratio for LM was 1:0.664, which can be inferred that the risk of transfer was 1.5 times higher in men than in women.

Considering the tremendous impact of the presence of LM on the prognosis of patients with osteosarcoma, it was necessary to screen outpatients with osteosarcoma at high risk of LM. The advantage of the nomogram was that clinicians could calculate the risk of LM more accurately with a simple piece of paper without having to understand complex formulas. The DCA has also proven its clinical usefulness, and if clinicians focused on DCA, they can make better medical decisions to the patient.

Although our study has several strengths, it also has several limitations. Firstly, this study was limited by the data collection of retrospective studies, which may have inherent biases. For example, the specifics of tumour composition and different surgical approaches were not available, which may lead to selection bias and limit the need for prospective data collection for further analysis.Secondly,although tumour size is often an important clinical predictive indicator in tumor disease, it was not found to be an independent risk factor associated with outcome in our study. Finally, although our nomogram was developed and validated in the SEER database and multicentre databases, prospective external validation of the prediction models is still necessary. Considering that all factors required in the nomograms is available clinical data, it can be used to effectively individualise the predictions. Of course, we hope to continuously improve the model in the future by incorporating a variety of other clinical factors to better facilitate clinicians

## Conclusions

In conclusion, our study was to develop a nomogram model on the prognosis of pulmonary metastases from osteosarcoma. We investigated a large number of patients based on the SEER database and a multicenter dataset. By analyzing the clinical characteristics and associated risk factors, we improve the prediction of lung metastasis risk and provide a basis for individualized treatment and follow-up strategies. The web-based calculator constructed in this study is an easy-to-use clinical tool that helps to promote personalized treatment.

## Data availability statement

Publicly available datasets were analyzed in this study. This data can be found here: Training set data for the model are available from the SEER database (https://seer.cancer.gov/data-software/), and validation set data sets generated and/or analyzed in the current study can be obtained from the corresponding authors upon reasonable request.

## Ethics statement

This study was approved by the Ethics Committee of Xianyang Central Hospital.

## Author contributions

CLY, WLL, TTW, and GYL designed the study. SPZ, LHC and JMW performed the study and analyzed the data. SPZ and HSW wrote the manuscript. ZHH, WYL and CX provided the expert consultations and clinical suggestions. All authors contributed to the article and approved the submitted version.
