# Supplementary material for: A clinical prediction model for lung metastasis risk in osteosarcoma: A multicenter retrospective study
Source: Front Oncol. 2023 Feb 10;13:1001219. doi: 10.3389/fonc.2023.1001219 (PMC9950508; doi:10.3389/fonc.2023.1001219)
Supplement: Supplementary file 1 [file Table_1.docx]

Table S1. Baseline table of patients with or without lung metastasis in the training group

| **Variables** | **level** | **Overall(772)** | **No(633)** | **Yes(139)** | **p** |
| --- | --- | --- | --- | --- | --- |
| Race (%) | black | 577 (74.7) | 472 (74.6) | 105 (75.5) | 0.812 |
|  | other | 123 (15.9) | 100 (15.8) | 23 (16.5) |  |
|  | white | 72 (9.3) | 61 (9.6) | 11 (7.9) |  |
| Age (mean (SD)) | NA | 32.90 (24.31) | 32.93 (24.04) | 32.76 (25.60) | 0.942 |
| Sex (%) | female | 433 (56.1) | 343 (54.2) | 90 (64.7) | 0.029 |
|  | male | 339 (43.9) | 290 (45.8) | 49 (35.3) |  |
| Primary.Site (%) | Axis bone | 500 (64.8) | 402 (63.5) | 98 (70.5) | 0.292 |
|  | Limb bone | 208 (26.9) | 177 (28.0) | 31 (22.3) |  |
|  | other | 64 (8.3) | 54 (8.5) | 10 (7.2) |  |
| Grade (%) | Moderately differentiated | 16 (2.1) | 16 (2.5) | 0 (0.0) | 0.206 |
|  | Poorly differentiated | 29 (3.8) | 25 (3.9) | 4 (2.9) |  |
|  | Undifferentiated; anaplastic | 183 (23.7) | 143 (22.6) | 40 (28.8) |  |
|  | unknown | 359 (46.5) | 295 (46.6) | 64 (46.0) |  |
|  | Well differentiated | 185 (24.0) | 154 (24.3) | 31 (22.3) |  |
| Laterality (%) | left | 347 (44.9) | 282 (44.5) | 65 (46.8) | 0.56 |
|  | Not a paired site | 326 (42.2) | 266 (42.0) | 60 (43.2) |  |
|  | right | 99 (12.8) | 85 (13.4) | 14 (10.1) |  |
| Stage.group (%) | I | 128 (16.6) | 127 (20.1) | 1 (0.7) | <0.001 |
|  | II | 356 (46.1) | 347 (54.8) | 9 (6.5) |  |
|  | III | 32 (4.1) | 31 (4.9) | 1 (0.7) |  |
|  | IV | 181 (23.4) | 54 (8.5) | 127 (91.4) |  |
|  | UNK stage | 75 (9.7) | 74 (11.7) | 1 (0.7) |  |
| T (%) | T1 | 267 (34.6) | 239 (37.8) | 28 (20.1) | <0.001 |
|  | T2 | 362 (46.9) | 293 (46.3) | 69 (49.6) |  |
|  | T3 | 21 (2.7) | 13 (2.1) | 8 (5.8) |  |
|  | TX | 122 (15.8) | 88 (13.9) | 34 (24.5) |  |
| N (%) | N0 | 703 (91.1) | 591 (93.4) | 112 (80.6) | <0.001 |
|  | N1 | 21 (2.7) | 13 (2.1) | 8 (5.8) |  |
|  | NX | 48 (6.2) | 29 (4.6) | 19 (13.7) |  |
| surgery (%) | No | 143 (18.5) | 90 (14.2) | 53 (38.1) | <0.001 |
|  | Yes | 629 (81.5) | 543 (85.8) | 86 (61.9) |  |
| Radiation (%) | No | 671 (86.9) | 559 (88.3) | 112 (80.6) | 0.021 |
|  | Yes | 101 (13.1) | 74 (11.7) | 27 (19.4) |  |
| Chemotherapy (%) | No | 167 (21.6) | 145 (22.9) | 22 (15.8) | 0.085 |
|  | Yes | 605 (78.4) | 488 (77.1) | 117 (84.2) |  |
| Bone.metastases (%) | No | 729 (94.4) | 616 (97.3) | 113 (81.3) | <0.001 |
|  | Yes | 37 (4.8) | 16 (2.5) | 21 (15.1) |  |
|  | unknown | 6 (0.8) | 1 (0.2) | 5 (3.6) |  |
